# Cost-effectiveness analysis of commercial Chinese polyherbal preparations for primary insomnia: based on network meta-analysis

**DOI:** 10.3389/fphar.2025.1682173

**Published:** 2025-12-08

**Authors:** Sihong Yang, Fanya Yu, Xinghua Xiang, Chuchuan Wan, Yumeng Tan, Ning Ma, Yajing Li, Long Ge, Xing Liao, Hui Zhao

**Affiliations:** 1 Institute of Basic Research In Clinical Medicine, China Academy of Chinese Medical Sciences, Beijing, China; 2 China Center for Evidence Based Traditional Chinese Medicine, Beijing, China; 3 Centre for Evidence Based Chinese Medicine, Beijing University of Chinese Medicine, Beijing, China; 4 The Research Center of National Drug Policy & Ecosysterm, China Pharmaceutical University, Nanjing, China; 5 School of Public Health, Lanzhou University, Lanzhou, China

**Keywords:** commercial Chinese polyherbal preparations, primary insomnia, pharmacoeconomic evaluation, cost-effectiveness analysis, network meta-analysis

## Abstract

**Objectives:**

Building upon prior systematic reviews and network meta-analyses, this study evaluated the cost-effectiveness of four commercial Chinese polyherbal preparations (CCPPs)—Tian Meng Oral Liquid/Capsules (TM), Shen-Qi-Wu-Wei-Zi Tablets (SQWWZ), Wu Ling Capsules (WL), and Bai-Le-Mian Capsules (BLM)—for treating primary insomnia. Comparative findings informed clinical decision-making and health policy formulation.

**Methods:**

A cost-effectiveness analysis was conducted from a healthcare system perspective. A patient disease course model was developed using systematic literature search and network meta-analysis combined with drug pricing data. Intervention strategies (monotherapy or combination therapy) were simulated to assess per-capita costs and health outcomes. Incremental cost-effectiveness ratios were calculated and compared against the willingness-to-pay threshold. Deterministic sensitivity analysis (DSA), Monte Carlo simulations for probabilistic sensitivity analysis and cost-effectiveness acceptability curves were used to test the overall stability and acceptable probability of the evaluation results.

**Results:**

At a WTP threshold of ¥89,358.00 per quality-adjusted life year (QALY), the combination of TM and benzodiazepines (BZDs) ranked highest in terms of cost-effectiveness, followed by WL, BLM, and SQWWZ. When the WTP threshold exceeded ¥5,897.63 per QALY, the probability that TM + BZDs was more cost-effective than WL increased. Likewise, when the WTP threshold was above ¥19,658.76 per QALY, the probability that BLM was more cost-effective than SQWWZ became greater.

**Conclusion:**

Within the 60-day time horizon of this analysis, TM + BZDs demonstrated optimal cost-effectiveness for primary insomnia from a healthcare system perspective, followed by WL and BLM. Limitations in data sources constrain the generalizability of findings. Future studies adopt a societal perspective and incorporate individual-level data over longer time horizons to validate and extend current results through more comprehensive cost-utility evaluations. Furthermore, research should focus on Traditional Chinese medicine (TCM)-specific health utility scales and informing equity-focused reimbursement policies to fully capture the value of CCPPs, thereby ultimately optimizing healthcare access and resource allocation.

## 1 Introduction

Insomnia is a prevalent sleep disorder. Primary insomnia (PI) is defined as a chronic condition lasting at least 3 months, marked by persistent sleep disturbances in the absence of underlying medical or psychiatric disorders (Sateia, 2014). PI is often attributed to intrinsic dysregulation of sleep mechanisms or abnormal cognitive-behavioral patterns, including neurotransmitter imbalances (e.g., serotonin, GABA), functional abnormalities in key brain regions (e.g., prefrontal cortex, hippocampus, thalamus), and disrupted functional connectivity within brain networks. Beyond reducing quality of life, PI is associated with an increased risk of comorbidities, such as anxiety, depression, hypertension, and cardiovascular diseases (Ge et al., 2019; Riemann et al., 2023). Epidemiological studies revealed that approximately 30%–36% of adults reported at least one nocturnal insomnia symptom (Morin and Jarrin, 2022), with 40% progressing to chronic insomnia (K Pavlova and Latreille, 2019) and nearly half of severe cases enduring for over a decade. Chronic insomnia incurs substantial economic burdens, including direct medical costs (e.g., medications, therapies) and indirect societal costs (e.g., productivity decline, accident risks) (Morin and Jarrin, 2022).

Pharmacotherapy remains the mainstay of treatment for primary insomnia, particularly in regions with limited access to cognitive behavioral therapy for insomnia (CBT-I) (Morin and Buysse, 2024; Riemann et al., 2023). This encompasses both conventional synthetic drugs and standardized herbal medicines. Common conventional pharmacological agents include BZDs, selective benzodiazepine receptor agonist (sBZRAs), and melatonin receptor agonists (e.g., ramelteon), which are preferred for their rapid efficacy. However, their prolonged use can lead to tolerance, dependence, and adverse effects such as cognitive impairment, nocturnal confusion, and falls (Barker et al., 2004; Stranks and Crowe, 2014; Treves et al., 2018). Additionally, discontinuation frequently triggers symptom rebound.

As a key component of herbal pharmacotherapy, Commercial Chinese polyherbal preparations (CCPPs) have garnered increasing attention for their unique theoretical framework and multifaceted therapeutic benefits in treating PI (Li et al., 2025; Xiong et al., 2024; Ye et al., 2024; Zhang et al., 2024). It is critical to note that the CCPPs evaluated in this study are China’s National Medical Products Administration (NMPA)-approved, standardized pharmacotherapies produced in compliance with the Chinese Pharmacopoeia. They are integral to the modernization of traditional Chinese medicine (TCM), adhering to stringent regulatory standards which ensure standardized compositions, dosing precision, and consistent therapeutic efficacy. By improving stability and reducing the burden of medication administration, CCPPs enhance the clinical utility of traditional formulations while preserving the holistic principles of TCM. The research team previously conducted a systematic review and network meta-analysis that evaluated the clinical efficacy and safety of CCPPs for the treatment of PI (Ma et al., 2025). The findings indicated that these medicines significantly improved sleep efficiency and quality compared to placebos and BZDs/sBZRAs, with favorable safety profiles. Four such CCPPs—Tian Meng Oral Liquid/Capsules (TM), Shen-Qi-Wu-Wei-Zi Tablets (SQWWZ), Wu Ling Capsules (WL), and Bai-Le-Mian Capsules (BLM)—are included in National Healthcare Security Administration (2022), with Wu Ling Capsules additionally included in the National Health Commission of the People’s Republic of China (2018). In addition to their regulatory endorsement, these CCPPs are among the most widely utilized treatments for primary insomnia in real-world clinical practice across China, routinely prescribed in both hospital and community settings. Detailed information regarding the formulation and manufacturing processes of the four CCPPs is provided in Table 1. Mechanistic studies of these medicines have revealed distinct therapeutic pathways: BLM regulated neurotransmitter levels and simultaneously influenced the gut microbiota (Bi, 2023); WL offered sedative, anxiolytic, and neuroprotective effects (You et al., 2021); TM regulated endocrine function and enhances cognitive performance (Liu X. Y., 2020); and SQWWZ stabilized the central nervous system and boosts immunity (Tan, 2015). Despite the promising therapeutic potential of CCPPs for PI management, critical knowledge gaps persist in regarding their comparative cost-effectiveness profiles. This is particularly salient for CCPPs with overlapping therapeutic indications that included in the China’s national insurance formulary or Essential Medicines List. Current decision-making processes lack robust pharmacoeconomic evidence to guide optimal medication selection for achieving dual synergistic policy goals: maximizing population health outcomes through cost-effective insomnia interventions while strategically expanding essential medicine coverage to enhance treatment accessibility.

**TABLE 1:** Basic information of 4 CCPPs.

Name	Formulation^&^	Manufacturer	Specification	Approval number	Preparation method^#^
Wu Ling capsules (WL)	Wuling Fungus Powder (330 g)	Zhejiang Jolly Pharmaceutical Co., Ltd.	0.33 g per capsule	National drug approval Z19990048	1. Fermentation of wuling fungus 2. Encapsulation of the resulting powder
Tian Meng oral liquid/Capsules (TM)	*Acanthopanax senticosus* (Rupr. and Maxim.) Harms (53 g), *Polygonatum sibiricum* Redouté (67 g), Bombycidae (13 g), *Morus alba* L. (33 g), *Codonopsis pilosula* Nannf. (40 g), *Astragalus mongholicus* Bunge (40 g), *Wurfbainia villosa* (lour.) Škorničk. and A.D.Poulsen (5 g), *Lycium barbarum* L. (40 g), *Crataegus pinnatifida* Bunge (160 g), Prepared Root of *Rehmannia chinensis* libosch. ex Fisch. and C.A.Mey. (27 g), *Epimedium brevicornu* Maxim. (27 g), *Citrus reticulata* Blanco (27 g), *Poria cocos* (Schw.)Wolf (27 g), *Strychnos nux-vomica* L. (1.3 g), *Pinellia ternata* (Thunb.) makino (27 g), *Alisma plantago-aquatica subsp. orientale* (Sam.) Sam. (40 g), *Dioscorea oppositifolia* L. (27 g)	Rongchang Pharmaceutical (Zibo) Co., Ltd.	10 mL per vial/0.4 g per capsule	National drug approval Z20153070/Z20153057	1. Water decoction of herbal mixture (twice) 2. Filtration and concentration 3. Ethanol precipitation and standing 4. Supernatant recovery, ethanol recovery, and concentration 5. Refrigeration, filtration, addition of preservative 6. Dilution, sterilization, and packaging
Bai-Le-Mian capsules (BLM)^a^	*Lilium brownii* var. *viridulum* Baker, *Acanthopanax senticosus* (Rupr. and Maxim.) Harms, *Reynoutria multiflora* (Thunb.) moldenke *Albizia julibrissin* var. *julibrissin*, mother-of-Pearl, Gypsum, *Ziziphus jujuba* var. *spinosa* (Bunge) Hu ex H.F.Chow, *Poria cocos* (Schw.)Wolf, *Polygala tenella* willd., *Scrophularia ningpoensis* Hemsl., *Rehmannia glutinosa* (Gaertn.) libosch. ex DC *Ophiopogon japonicus* (Thunb.) Ker Gawl *Schisandra chinensis* (Turcz.) Baill., *Juncus conglomeratus* L *Salvia miltiorrhiza* Bunge. Excipient: Starch	Yangtze River Pharmaceutical group Co., Ltd.	0.27 g per capsule	National drug approval Z20020131	Unavailable
Shen-Qi-Wu-Wei-Zi Tablets (SQWWZ)	*Kadsura longipedunculata* Finet and Gagnep. (180 g), *Codonopsis pilosula* Nannf. (60 g), *Astragalus mongholicus* Bunge (120 g), *Ziziphus jujuba* var. *spinosa* (Bunge) Hu ex H.F.Chow (30 g) Excipient: Starch, Sugar powder, Talcum powder	Kangxian Duyiwei Biopharmaceutical Co., Ltd.	0.26 g per tablet	National drug approval Z20103006	1. Grinding: Two herbs ground into powder 2. Ethanol maceration: Three herbs separately macerated with ethanol at varying concentrations for 24 h 3. Percolate collection and ethanol recovery 4. Concentration of the percolates 5. Mixing with powder and excipients 6. Granulation, drying, and compression into tablets

^a^ Dosage details for Bai-Le-Mian Capsules’ composition are unavailable; ^&^The botanical components in the formulations have all been verified against The World Flora Online (https://www.worldfloraonline.org/)[2025-2-25]; ^#^Preparation methods sourced from Chinese Pharmacopoeia 2020 Edition (Chinese Pharmacopoeia Commission, 2020) and Yaozhi Database (https://www.yaozh.com). For detailed technical parameters, see Supplementary Table S1.

This study conducted a comparative cost-effectiveness analysis of four insurance-covered CCPPs for PI, integrating systematic reviews, network meta-analysis, and pharmacoeconomic modeling. The findings aimed to support the development of cost-effective, safety-optimized insomnia treatments, establish an evidence base for refining healthcare reimbursement frameworks and essential medicine list revisions, and further promote the global standardization of CCPPs through clinical-economic evidence synthesis (Xiong et al., 2022).

## 2 Methods

### 2.1 Evaluation and reporting standards

The economic evaluation was conducted in accordance with the Liu G. E. (2020), Guidelines for Comprehensive Clinical Evaluation of Drugs (2021), and Guidelines for Clinical Comprehensive Evaluation of Commercial Chinese polyherbal preparations (2022) (Yuan et al., 2023). Reporting of results followed the Consolidated Health Economic Evaluation Reporting Standards 2022 (Cheers 2022) (Husereau et al., 2022), with the full checklist available in the Supplementary Table S2.

### 2.2 Source of clinical efficacy data

#### 2.2.1 Data source

The clinical efficacy data for this analysis were sourced from a previously published systematic review and network meta-analysis (Ma et al., 2025). By synthesizing evidence from 45 interventions and 109 randomized controlled trials, this NMA directly addressed the comparative efficacy of the interventions of interest, precluding the risk of combining data from multiple inconsistent sources and ultimately providing a robust and coherent evidence base for the economic model.

The source NMA strictly adhered to the PRISMA-Network Meta-Analysis extension statement (Hutton et al., 2015). A comprehensive and systematic search was conducted across eight databases. It employed a random-effects model for analysis, assessed inconsistency using the node-splitting method, ensuring the reliable results. Methodological quality of the included RCTs was assessed using a modified version of the Cochrane Risk of Bias tool (Partners, 2011).

#### 2.2.2 Inclusion and exclusion criteria

To better align with clinical needs and healthcare insurance policies, this study defined inclusion and exclusion criteria to further screen the literature pool included in the original network meta-analysis.

##### 2.2.2.1 Inclusion criteria

- Types of Studies: Only randomized controlled trials were selected for inclusion. Trials were required to report adequate randomization procedures and provide sufficient methodological information to allow assessment of risk of bias.
- Types of Population: The study population consisted of patients diagnosed with primary insomnia according to internationally recognized criteria (e.g., DSM-III, DSM-III-R, DSM-IV, DSM-IV-TR, DSM-5).
- Types of Interventions: Interventions were required to be CCPPs with sedative effects that are listed in National Healthcare Security Administration (2022) or the National Health Commission of the People’s Republic of China (2018).
- Types of Outcomes: The primary outcome was sleep quality, as assessed by the Pittsburgh Sleep Quality Index (PSQI). Studies were required to report baseline and post-treatment PSQI scores or provide sufficient data for effect size calculation.

##### 2.2.2.2 Exclusion criteria

Studies were excluded if they exhibited substantial disparities in drug composition, route of administration, or dosage form compared to other interventions.

### 2.3 Cost-effectiveness analysis

#### 2.3.1 Overview

The study adopted a healthcare system perspective to simulate patient disease progression and perform a cost-effectiveness analysis (CEA). Treatment processes for insomnia patients under different intervention and control regimens were modeled to estimate *per capita* costs and health outcomes. The incremental cost-effectiveness ratio (ICER) was calculated and compared against the willingness-to-pay (WTP) threshold. According to the Liu G. E. (2020), the WTP threshold was typically set at one to three times the *per capita* GDP. To more conservatively and sensitively assess cost-effectiveness, this study adopted a WTP threshold equal to one times China’s *per capita* GDP in 2023 (¥89,358). All costs in this study were expressed in 2023 Chinese Yuan (CNY).

The study duration was determined by comprehensively considering both the Chinese insomnia diagnosis and treatment guidelines (Chinese Sleep Research Society, 2023) and the treatment durations reported in the included studies.

Due to data availability constraints, individual-level recovery trajectory data were not obtainable. Therefore, based on existing research evidence and guideline recommendations, this study modeled a linear recovery from treatment completion until the end of the study time frame, with all patients reaching a normal health state by that endpoint. This approach could potentially favor treatments with shorter durations, as they may gain higher QALY values during the post-treatment recovery period. However, the study considered this to reflect the inherent benefit of faster-acting therapies or those requiring shorter treatment durations, constituting a legitimate benefit.

#### 2.3.2 Model

**FIGURE 1:**
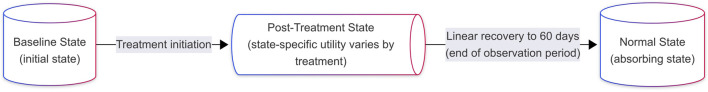
Model state transition diagram.

A model with three health states was constructed: baseline state, post-treatment state, and normal state (Figure 1). The baseline state served as the initial entry point, while the normal state was defined as the absorbing state. Health utility values for the baseline and normal states were obtained from previously published literature (Liao et al., 2022; Wen et al., 2021). However, due to the lack of individual-level data on complete recovery at the end of treatment, only the average Pittsburgh Sleep Quality Index (PSQI) score and its corresponding utility value at that time point were available.

To better approximate real-world recovery patterns and considering the chronic nature of insomnia, the model assumed that all patients would recover linearly to the normal health state by the end of the treatment observation period.

As a result, transitions between health states were modeled as unidirectional: from the baseline state to the post-treatment state, and then to the normal state. Differences between treatment regimens were reflected in the timing of transitions and the utility values associated with the post-treatment state.

#### 2.3.3 Costs

The cost analysis was restricted to drug acquisition costs. These costs were calculated using Formula 1, which incorporating unit price, daily dosage, and treatment duration. Unit prices were derived from the 2023 average tender prices in the MENET database (https://db.menet.com.cn/#/). The weighted daily dosage was determined through meta-analysis of dosages reported in included studies, with weights assigned based on study sample sizes. Similarly, the weighted treatment duration was calculated using the same methodological approach. The cost of additional medication required to manage severe adverse events was included in the total cost. Conversely, no adverse event costs were calculated for self-limiting events or if no such events occurred (Liu G. E., 2020).

$$Total\;Drug\;Cost=\sum \left(Unit\;Price\times Daily\;Dose\times Treatment\;Duration\right)$$ (1)

#### 2.3.4 Effectiveness

Health outcomes were quantified using quality-adjusted life years (QALYs), calculated through the following steps.

1. Baseline and post-treatment PSQI scores were derived from a prior network meta-analysis (Ma et al., 2025). All patients were assumed to linearly transition to the Normal State post-treatment, given comparable baseline characteristics across studies.
2. Health utility changes were estimated based on published evidence linking PSQI reductions to EuroQol Five Dimensions Questionnaire (EQ-5D) utility values in Chinese populations (Liang et al., 2022), where a 1 standard deviation (SD) improvement in PSQI corresponded to a 0.0046 utility gain. Baseline (0.624) and Normal State (0.964) utilities were sourced from published pharmacoeconomic studies (Wen et al., 2021) and large-scale cross-sectional data (Liao et al., 2022) specific to Chinese insomnia patients.
3. QALYs were calculated by integrating PSQI score trajectories (Formula 2), time-dependent utility changes (Formula 3), and cumulative health gains over the observation period (Formula 4).

PSQI_regimen=PSQI_reference+PSQI_difference (2)

$$\text{Utility}\_\left(post-treatment\right)=\text{Utility}\_baseline+\text{PSQI}\_reduction\times \Delta \text{Utility}\_\left(per\;unit\;PSQI\;reduction\right)$$ (3)

$$\begin{array}{c} QALY=\left({Utility}_{normal}+{Utility}_{baseline}\;\right)\times Treatment\;Duration÷2÷365\\ +\left(\text{Utility}\_normal+\text{Utility}\_\left(post-treatment\right)\right)\\ \times \left(Study\;Duration-Treatment\;Duration\right)÷2÷365 \end{array}$$ (4)

*QALYs were estimated in two segments: from the baseline state to the post-treatment state, and from the post-treatment state to the normal state, corresponding to the two distinct transition phases in the model. A visual illustration of this two-phase QALY estimation model is provided in Supplementary Figure S1.

#### 2.3.5 Sensitivity analysis

To validate the robustness of the findings, sensitivity analysis was conducted. First, a deterministic sensitivity analysis (DSA) was conducted to identify the key drivers of model uncertainty. Each parameter was varied individually across its plausible range (minimum to maximum values) while holding all others constant. The results are presented as tornado diagrams, which rank parameters by their relative influence on the ICER. Second, the probabilistic sensitivity analysis (PSA) was employed to quantify the overall decision uncertainty by simultaneously accounting for the joint uncertainty in all model parameters. 10,000 Monte Carlo simulations were conducted with parameter values drawn from their pre-specified statistical distributions. For each simulation, incremental costs and QALYs were calculated. The results of the PSA were used to construct cost-effectiveness acceptability curves (CEACs), which estimate the probability of each regimen being economically preferable across WTP thresholds ranging from ¥0 to ¥200,000 per QALY. As baseline health status, normal state definitions, and the utility gain per unit reduction in PSQI score were consistent across all treatment strategies, the baseline utility value, utility gain per unit PSQI improvement, and utility value for the normal state were treated as fixed parameters. Other variables, including treatment duration, dosage regimens, drug prices, and differences in PSQI scores between strategies, were modeled using gamma distributions for PSA. The uncertainty in the efficacy parameters (PSQI score differences), captured by these distributions, is a major source of uncertainty in the model’s outcomes.

To assess the robustness of the base-case findings, scenario analyses were performed to test key structural and cost assumptions. Alternative Recovery Scenarios: The base-case linear recovery assumption was tested by (1) extending the recovery duration to 90 days, and (2) assuming only 70% of patients achieved full recovery. Inclusion of Medical Consultation Costs: A scenario incorporating standard outpatient consultation fees (¥20 per visit) was conducted, with visit frequency aligned to the recommended physician visits schedule for each treatment regimen.

## 3 Results

### 3.1 Clinical efficacy evidence from the NMA

#### 3.1.1 Literature screening

Of the initial 109 RCTs from the source NMA (Ma et al., 2025), 38 were excluded as their investigated interventions did not meet the inclusion criteria. Following a title and abstract screening of the remaining 71 studies, 52 were excluded due to irrelevance. Of the 19 studies left, 4 were excluded after full-text review. Ultimately, 15 RCTs were included in the final analysis. Four preparations were involved: Tian Meng Oral Liquid/Capsules, Shen-Qi-Wu-Wei-Zi Tablets, Wu Ling Capsules, and Bai-Le-Mian Capsules. The study selection process was summarized in the PRISMA flow diagram (Supplementary Figure S2).

#### 3.1.2 Characteristics of the efficacy data

A total of 15 RCTs were included in this analysis. The investigated regimens encompassed 7 trials of WL, 3 trials of BLM, 3 trials of a TM + BZDs, and 2 trials of SQWWZ. The basic characteristics of the included literature were presented in the Supplementary Table S3. The estimated differences in PSQI score reductions between the various treatment regimens were presented in Supplementary Table S4.

Given that TM is frequently combined with BZDs (e.g., oxazepam) in clinical practice, the TM + BZDs was analyzed as a distinct intervention. Other CCPPs were evaluated as monotherapies. Six intervention-control combinations were established (Table 2).

**TABLE 2:** Intervention and control regimen combinations.

Combination	Intervention regimen	Control regimen
1	TM + BZD	WL
2	TM + BZD	SQWWZ
3	TM + BZD	BLM
4	WL	SQWWZ
5	WL	BLM
6	SQWWZ	BLM

TM: Tian Meng Oral Liquid/Capsules; BZDs: benzodiazepines; WL: wu ling capsules; BLM: Bai-Le-Mian; SQWWZ: Shen-Qi-Wu-Wei-Zi Tablets.

#### 3.1.3 Methodological quality

The methodological quality of the included RCTs was assessed using an adapted version of the ROB 2.0 tool (Partners, 2011). The complete results of the risk of bias assessment for all included studies were presented in Supplementary Table S5.

### 3.2 Cost-effectiveness analysis results

#### 3.2.1 Base-case analysis

##### 3.2.1.1 Model Parameters and Assumptions

Detailed specifications of all model parameters are provided in Table 3. The study employed a 60-day observation period following treatment initiation. This duration was chosen to encompass the full range of treatment durations (28–56 days) reported in the included studies, allowing for a comprehensive assessment of each CCPPs’ effectiveness and enabling direct comparability between them. That also took into consideration the recommendations for ongoing assessments during insomnia therapy outlined in the Chinese insomnia diagnosis and treatment guidelines (Chinese Sleep Research Society, 2023). Given the short timeframe (below the threshold requiring discounting per Chinese guidelines), no discounting was applied to costs or health outcomes.

**TABLE 3:** Detailed model parameters.

Data type	Data label	Model input value	Mean	Min	Max	Simulated value	Std. Dev	Std. Error	Distribution type
Effectiveness	PSQl score reduction: SQWWZ	3.602913	3.602913	3.5	3.7	3.6531	0.100014	0.057743	Gamma
	Reduction difference in PSQl scores: SQWWZ vs. TM + BZ	0.16	0.16	−4.65	4.97	0.0000	4.81	2.777055	Gamma
	Reduction difference in PSQI scores: SQWWZ vs. WL	0.79	0.79	−3.64	5.22	0.0000	4.43	2.557662	Gamma
	Reduction difference in PSQl scores: SQWWZ vs. BLM	1.84	1.84	−2.95	6.63	0.3719	4.79	2.765508	Gamma
Utility	Utility value in baseline state	0.624	0.624	0.624	0.624	0.6240	0	0	Fixed
	Utility gained per unit PSQI reduction	0.0046	0.0046	0.0046	0.0046	0.0046	0	0	Fixed
	Utility value in normal state	0.964	0.964	0.964	0.964	0.964	0	0	Fixed
Medication	Weighted average daily dosage of SQWWZ	15	15	13.5	16.5	15.37919	1.5	0.866025	Gamma
	Weighted average treatment duration of SQWWZ	56	56	50.4	60	54.40357	4.822171	2.784082	Gamma
	Weighted average daily dosage of TM oral liquid	2	2	1.8	2.2	1.890147	0.2	0.11547	Gamma
	Weighted average treatment duration of TM oral liquid	28	28	25.2	30.8	29.33514	2.8	1.616581	Gamma
	Weighted average daily dosage of TM Capsule	6	6	5.4	6.6	6.584417	0.6	0.34641	Gamma
	Weighted average treatment duration of TM Capsule	28	28	25.2	30.8	31.08172	2.8	1.616581	Gamma
	Weighted average daily dosage of WL	8.808	8.808	7.9272	9.6888	8.983993	0.8808	0.50853	Gamma
	Weighted average treatment dosage of WL	30.13333	30.13333	27.12	33.14667	27.65636	3.013333	1.739749	Gamma
	Weighted average daily dosage of BLM	8	8	7.2	8.8	8.232057	0.8	0.46188	Gamma
	Weighted average treatment dosage of BLM	35.97927	35.97927	32.38135	39.5772	38.21427	3.597927	2.077264	Gamma
	Weighted average daily dosage of BZD	1.5	1.5	1.35	1.65	1.488102	0.15	0.086603	Gamma
	Weighted average treatment dosage of BZD	28	28	25.2	30.8	26.72673	2.8	1.616581	Gamma
	Average winning bid price per unit of BLM	0.392407	0.392407	0.36	0.4278	0.357093	0.033911	0.019579	Gamma
	Average winning bid price per unit of TM oral liquid	2.933275	2.933275	2.9306	2.97	2.923672	0.022016	0.012711	Gamma
	Average winning bid price per unit of TM Capsule	0.784613	0.784613	0.7222	0.8306	0.821078	0.054407	0.031412	Gamma
	Average winning bid price per unit of WL	1.064196	1.064196	0.8568	1.2422	1.156851	0.192887	0.111363	Gamma
	Average winning bid price per unit of BLM	1.741186	1.741186	1.32	1.7708	1.852112	0.252156	0.145582	Gamma
	Average winning bid price per unit of BZD	3.139	3.139	2.8251	3.4529	3.2914	0.3139	0.18123	Gamma

PSQI: pittsburgh sleep quality index; SQWWZ: Shen-Qi-Wu-Wei-Zi Tablets; TM: Tian Meng Oral Liquid/Capsules; BZDs: benzodiazepines; WL: wu ling capsules; BLM: Bai-Le-Mian Capsules; Std. Dev: Standard Deviation; Std. Error: Standard Error.

##### 3.2.1.2 Base-Case Cost and Effectiveness Outcomes

Direct medical costs were limited to drug acquisition costs, as previous studies evaluating the included medications reported only mild adverse events (e.g., mild gastrointestinal reactions, dizziness) (Ma et al., 2025). These events typically resolved spontaneously after treatment discontinuation and did not result in additional healthcare resource utilization (e.g., outpatient visits, emergency care, or hospitalization); therefore, costs associated with adverse drug reactions were not considered in the analysis (Liu G. E., 2020). The relevant cost calculation results were presented in Table 4.

**TABLE 4:** Cost calculation gains by regimen.

Regimen	Medications	Daily dosage	Duration(d)	Unit prices (¥)	Medication cost (¥)	Total regimen cost (¥)
SQWWZ	SQWWZ	15.000	56.000	0.392	329.622	329.622
TM + BZDs	TM (oral Liquid)	2.000	28.000	2.933	164.263	287.812
	TM (Capsules)	6.000	28.000	0.785	131.815	
	BZD	1.500	28.000	3.139	131.838	
WL	WL	8.808	30.133	1.064	282.453	282.453
BLM	BLM	8.000	35.979	1.741	501.173	501.173

SQWWZ: Shen-Qi-Wu-Wei-Zi Tablets; TM: Tian Meng Oral Liquid/Capsules; BZDs: benzodiazepines; WL: wu ling capsules; BLM: Bai-Le-Mian Capsules.

This study derived the relative differences in PSQI score reductions from the network meta-analysis (Supplementary Table S4). The absolute PSQI reduction for SQWWZ (3.603), serving as the benchmark, was calculated as a weighted average from the literature. This benchmark was then used to compute the absolute reductions for other interventions, which in turn informed the calculation of health state utilities and QALYs for all treatment regimens, with full results presented in Table 5.

**TABLE 5:** Health utilities and QALY gains by regimen.

Regimen	PSQI reduction	Utility				QALY		
		Utility gain	Baseline utility	Post-treatment utility	Normal utility	QALY during treatment	QALY during recovery	Total QALY
SQWWZ	3.603	0.017	0.624	0.641	0.964	0.097	0.009	0.106
TM + BZD	3.443	0.016	0.624	0.640	0.964	0.048	0.070	0.119
WL	2.813	0.013	0.624	0.637	0.964	0.052	0.065	0.118
BLM	1.763	0.008	0.624	0.632	0.964	0.062	0.053	0.114

PSQI: Pittsburgh sleep quality index; QALY: quality-adjusted life year; SQWWZ: Shen-Qi-Wu-Wei-Zi Tablets; TM: Tian Meng Oral Liquid/Capsules; BZDs: benzodiazepines; WL: Wu ling capsules; BLM: Bai-Le-Mian Capsules.

##### 3.2.1.3 Incremental Cost-Effectiveness Results

The cost-effectiveness analysis (CEA) results for the six treatment comparisons were presented in Table 6. In the comparison between the TM + BZDs regimen and the WL regimen, the TM + BZDs regimen incurred a total treatment cost of ¥287.812 and yielded 0.119 QALYs, while the WL regimen incurred ¥282.453 and yielded 0.118 QALYs. This resulted in a QALY gain of 0.001 at an incremental cost of ¥5.359, producing an ICER of ¥4,350.317 per QALY gained. To contextualize this QALY gain, 0.001 QALYs over 60 days is equivalent to approximately 0.06 days, or about 8.76 h, of perfect health. Within the 60-day time horizon of this analysis, and as this value was below the WTP threshold of ¥89,358 (equivalent to China’s 2023 *per capita* GDP), the TM + BZDs regimen was considered cost-effective compared with the WL regimen.

**TABLE 6:** Cost-effectiveness analysis results.

Combination	Regimen	QALY	Costs (¥)	ΔQALY	ΔCost (¥)	ICER (¥/QALY)	WTP (¥)	Dominance
1	TM + BZD	0.119	287.812	0.001	5.359	4350.317	89,358	Dominant
	vs. WL	0.118	282.453					
2	TM + BZD	0.119	287.812	0.013	−41.81	−3220.957	89,358	Dominant
	vs. SQWWZ	0.106	329.622					
3	TM + BZD	0.119	287.812	0.004	−213.361	−49031.042	89,358	Dominant
	vs. BLM	0.114	501.173					
4	WL	0.118	282.453	0.012	−47.169	−4014.764	89,358	Dominant
	vs. SQWWZ	0.106	329.622					
5	WL	0.118	282.453	0.003	−218.72	−70108.062	89,358	Dominant
	vs. BLM	0.114	501.173					
6	SQWWZ	0.106	329.622	−0.009	−171.551	19,880.658	89,358	Dominated
	vs. BLM	0.114	501.173					

QALY: quality-adjusted life year; ICER: incremental cost-effectiveness ratio; WTP: willingness-to-pay; TM: Tian Meng Oral Liquid/Capsules; BZDs: benzodiazepines; WL: Wu ling capsules; SQWWZ: Shen-Qi-Wu-Wei-Zi Tablets; BLM: Bai-Le-Mian Capsules.

#### 3.2.2 Sensitivity analysis

##### 3.2.2.1 Deterministic Sensitivity Analysis

**FIGURE 2:**
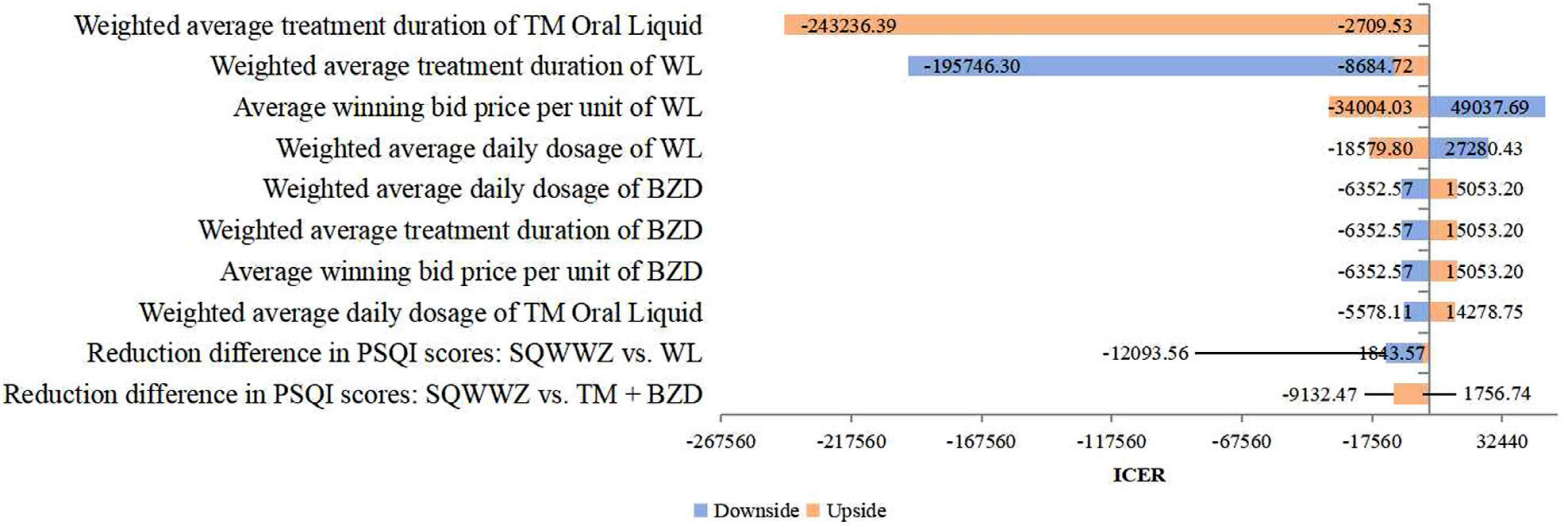
Tornado Diagram for the Cost-efectiveness Analysis of TM + BZDs vs. WL in the Deterministic Sensitivity Analysis.

**FIGURE 3:**
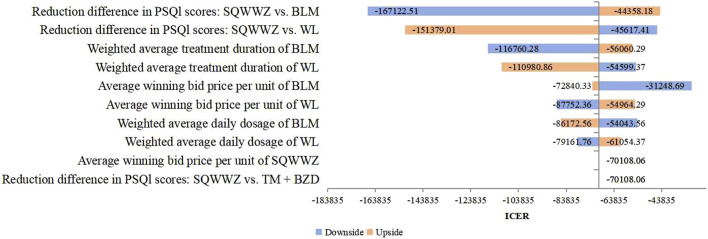
Tornado Diagram for the Cost-efectiveness Analysis of WL vs. BLM in the Deterministic Sensitivity Analysis.

**FIGURE 4:**
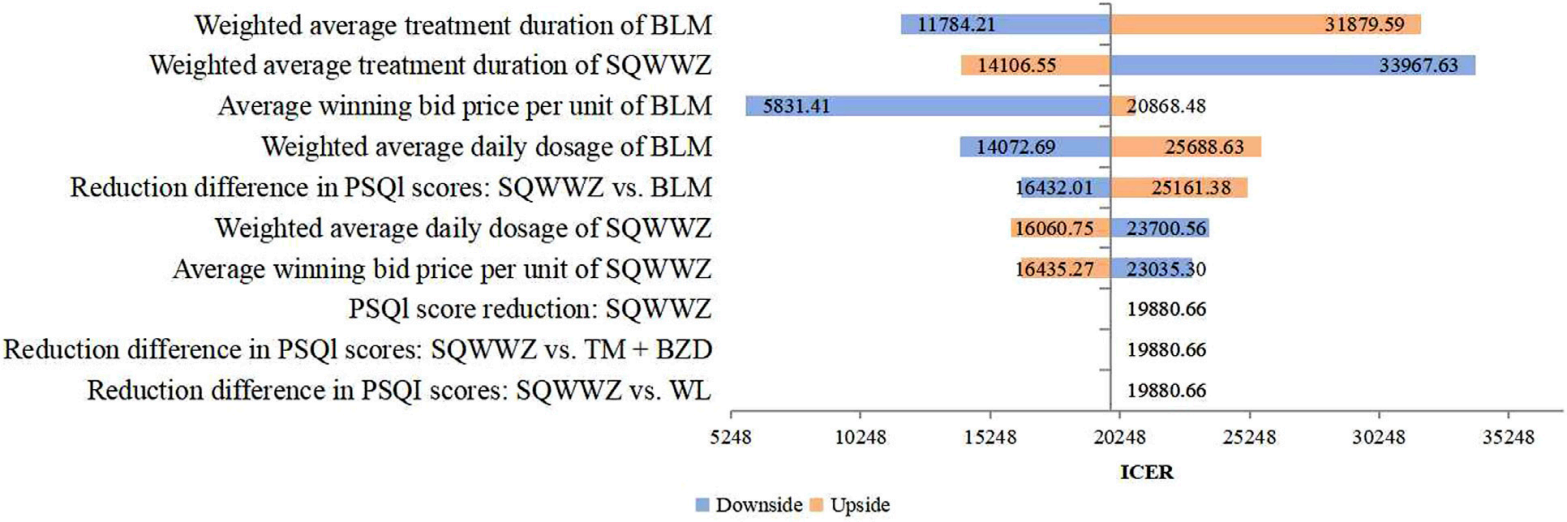
Tornado Diagram for the Cost-efectiveness Analysis of SQWWZ vs. BLM in the Deterministic Sensitivity Analysis.

**FIGURE 5:**
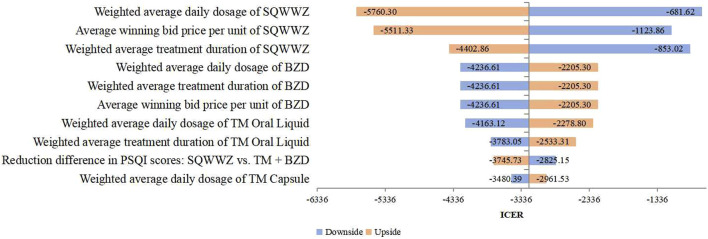
Tornado Diagram for the Cost-efectiveness Analysis of TM + BZDs vs. SQWWZ in the Deterministic Sensitivity Analysis.

Deterministic sensitivity analysis was conducted on key parameters, such as treatment protocols and PSQI reduction values. For the TM + BZDs versus WL comparison, Figure 2 identified the 10 most influential parameters affecting the ICER, with the weighted average treatment duration of TM and WL emerging as the two dominant drivers. Under these parameter variations, the ICER ranged from ¥–243,236.39 per QALY to ¥49,037.69 per QALY. In the comparison between the WL and BLM regimen, the 10 most influential parameters affecting the ICER were presented in Figure 3. The two parameters with the largest impact were the difference in PSQI score reductions between the SQWWZ and BLM regimens, and between the SQWWZ and WL regimens. The ICER fluctuated within a range of ¥–167,122.51 per QALY to ¥–31,248.69 per QALY under these parameter variations. Tornado diagrams for other regimen comparisons were shown in Figures 4, 5.

##### 3.2.2.2 Probabilistic Sensitivity Analysis and Cost-Effectiveness Acceptability

**FIGURE 6:**
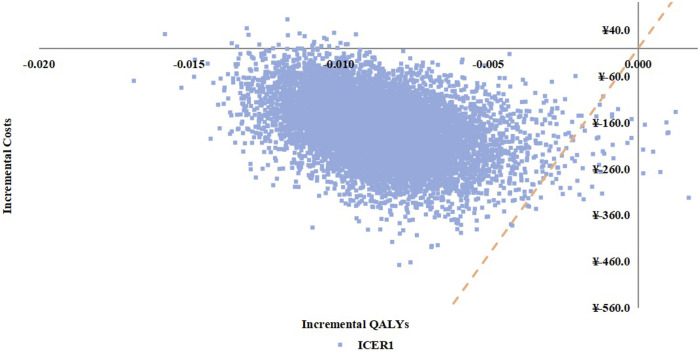
Ineremental Cost-effectivenes Ratio Scatter Plot for SQWWZ vs. BLM in the Probabilistic Sensitivity Analysis.

The probabilistic sensitivity analysis (PSA) revealed that SQWWZ demonstrated a 0.57% probability of cost-effectiveness dominance compared to BLM. Cost-effectiveness probabilities for other regimen pairs were summarized in Table 7. The scatter plot of ICERs for SQWWZ vs. BLM was provided in Figure 6.

**TABLE 7:** Probability of cost-effectiveness dominance for each regimen pair.

Combination	Regimen pair	Probability of dominance
1	TM + BZD vs. WL	76.88%
2	TM + BZD vs. SQWWZ	98.39%
3	TM + BZD vs. BLM	98.34%
4	WL vs. SQWWZ	99.93%
5	WL vs. BLM	98.93%
6	SQWWZ vs. BLM	0.57%

TM: Tian Meng Oral Liquid/Capsules; BZDs: benzodiazepines; SQWWZ: Shen-Qi-Wu-Wei-Zi Tablets; WL: Wu ling capsules; BLM: Bai-Le-Mian Capsules.

**FIGURE 7:**
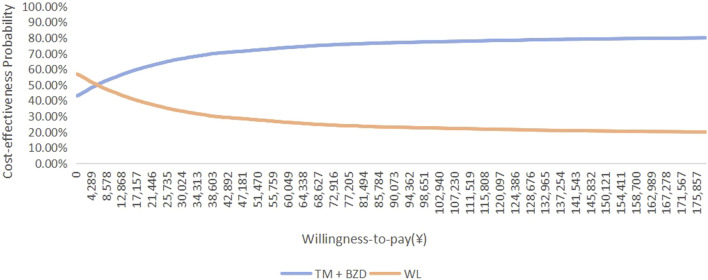
Cost-effectiveness acceptability curves of TM + BZDs and WL in a probabilistic sensitivity analysis.

**FIGURE 8:**
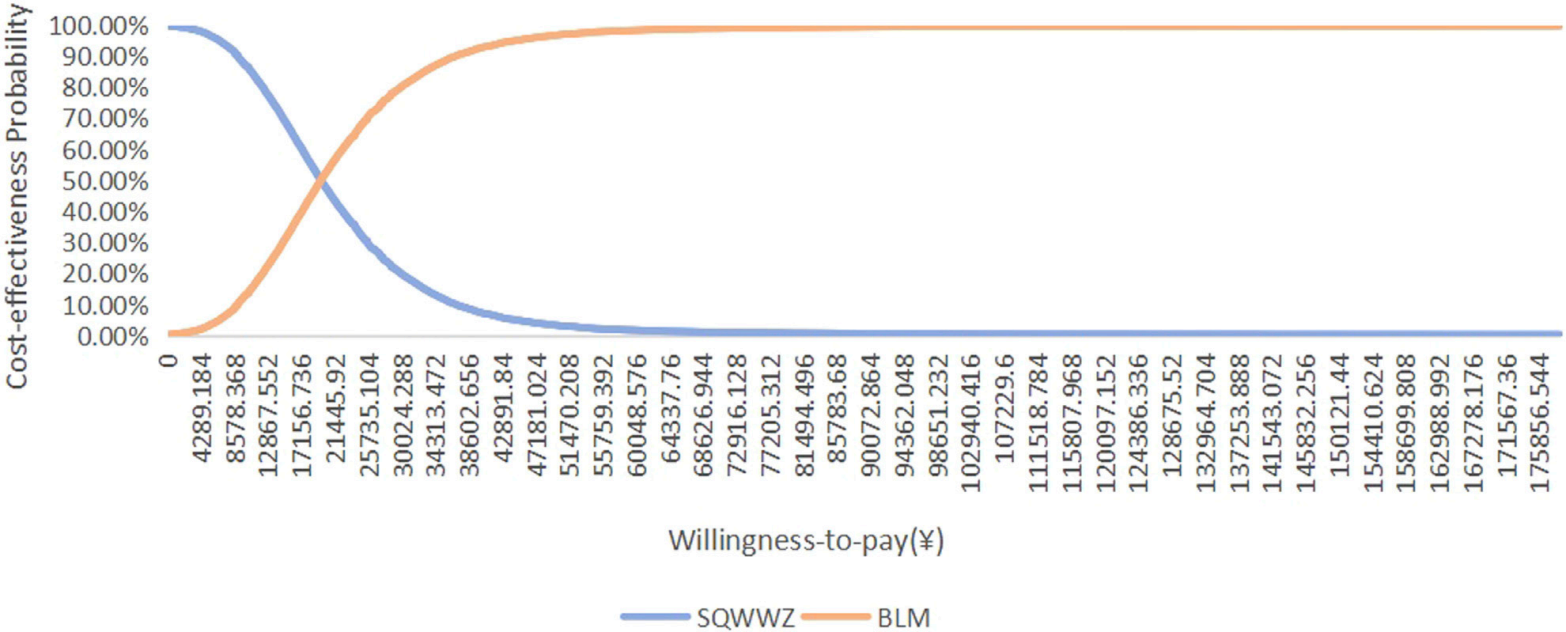
Cost-effectiveness acceptability curves of SQWWZ and BLM in a probabilistic sensitivity analysis.

Cost-effectiveness acceptability curves demonstrated threshold-dependent preferences. When the WTP threshold exceeded ¥5,897.63 per QALY, the probability that the TM + BZDs regimen was more cost-effective than the WL regimen increased, as illustrated in Figure 7. When the WTP threshold was greater than ¥19,658.76 per QALY, the probability that the BLM regimen was more cost-effective than the SQWWZ regimen increased (Figure 8). Separately, it was observed that the SQWWZ regimen was associated with a greater reduction in PSQI score but also required a longer treatment duration (56 days) compared to BLM (35.98 days). Regardless of the WTP threshold, TM + BZDs consistently demonstrated cost-effectiveness dominance over both the SQWWZ and BLM, and WL maintained dominance over both SQWWZ and BLM. These findings underscored the robust economic value of optimized combination therapies in insomnia management under diverse reimbursement policies.

##### 3.2.2.3 Scenario Analyses

The results of the scenario analyses, detailed in Supplementary Table S6, confirmed the robustness of the base-case findings. Under alternative recovery assumptions—including prolonged duration and incomplete recovery—the cost-effectiveness ranking remained unchanged, with TM + BZDs consistently yielding the most favorable ICER. Similarly, the incorporation of medical consultation costs did not alter the hierarchical order of the regimens. The stability of the results across all scenarios underscores the reliability of the primary conclusion.

## 4 Discussion

This comparative cost-effectiveness evaluation of four CCPPs showed that, at a WTP threshold of ¥89,358.00 per QALY, and within the 60-day time horizon, the combination of TM + BZDs was the most cost-effective regimen for primary insomnia. Base-case analysis demonstrated a consistent ranking: TM + BZDs yielded the most cost-effective option, followed by WL, BLM, and SQWWZ. Probabilistic sensitivity analysis confirmed the robustness of this overall hierarchy while revealing important nuances in the SQWWZ-BLM comparison. Despite SQWWZ’s superior efficacy in PSQI reduction, its cost-effectiveness advantage was unstable, with only a 0.57% probability of dominance over BLM. Cost-effectiveness acceptability curves further demonstrated that BLM becomes the preferred option when the willingness-to-pay threshold exceeds ¥19,658.76 per QALY. This counterintuitive finding result can be explained by the model structure, which comprises two distinct health states: “treatment” and “recovery”. SQWWZ’s longer treatment duration (56 days vs. BLM’s 35.98 days) delays transition to the higher-utility recovery state. As a result, SQWWZ accumulates fewer QALYs despite its greater symptomatic efficacy. This demonstrates how treatment duration can outweigh incremental efficacy gains in short-term cost-utility frameworks.

This analysis shows that combining a CCPPs with BZDs constitutes a cost-effective pharmacological strategy for the short-term management of primary insomnia. This finding contributes to a still-maturing body of pharmacoeconomic evidence for insomnia, which has thus far focused predominantly on newer-generation prescription agents. Indeed, recent cost-effectiveness analyses have substantiated the value of modern, targeted pharmacotherapies—including daridorexant (Briggs et al., 2025), suvorexant (Nishimura and Nakao, 2018), and lemborexant (Ikeda et al., 2022). Conducted across diverse healthcare systems, these studies consistently report that such agents are cost-effective, or even cost-saving, compared to older-generation alternatives like zolpidem, particularly when modeling broader outcomes such as fracture risk.

The present study broadens the scope of insomnia pharmacoeconomics in two pivotal respects. First, it deliberately shifts the evaluative focus from high-cost, patented pharmaceuticals to standardized, approved herbal pharmacotherapies—interventions rooted in a distinct therapeutic paradigm and cost structure. Second, it moves beyond the prevailing literature’s direct comparison of novel versus conventional prescription drugs. Instead, it demonstrates the economic value of integrating herbal pharmacotherapy into conventional treatment protocols, as exemplified by the TM + BZDs combination.

The demonstrated economic competitiveness of this CCPPs-based regimen substantially enlarges the range of cost-effective options available to clinicians and payers. It compellingly indicates that the efficient management of insomnia need not be the exclusive purview of the latest patented drugs. Significant value can also be realized through the strategic deployment of established herbal pharmacotherapy within evidence-based, integrated treatment pathways.

### 4.1 Study limitations

While this study provided a pharmacoeconomic evaluation of oral CCPPs for primary insomnia, several limitations must be acknowledged.

First, the limited availability of primary pharmacoeconomic studies directly comparing these regimens necessitated the use of efficacy data derived from published systematic reviews and network meta-analyses. This approach may have introduced inherent heterogeneity into the model’s input parameters, particularly in baseline health utilities and PSQI-to-utility conversion coefficients.

Second, utility data for baseline and normal states were sourced from different published studies constrained by data availability. However, the studies by Wen and Liao (Liao et al., 2022; Wen et al., 2021) were both conducted among Chinese patients with insomnia and were thus consistent with the target population of this study. Specifically, the baseline utility value was taken from Wen (Wen et al., 2021), in which patients had not received any intervention at the time of assessment—making the estimate representative of the untreated baseline utility for Chinese insomnia patients in this study. The utility value for normal state was sourced from Liao (Liao et al., 2022), which assessed utility among Chinese individuals with good sleep quality, providing a reasonable proxy for the normal state in the model. Nevertheless, it must be acknowledged that utility values derived from different sources may be affected by variations in patient baseline characteristics, which could introduce bias into the model results.

Third, the limited quality of the available studies likely exacerbated the heterogeneity, further influencing the robustness of the findings. The statistical heterogeneity of NMA was assessed using Cochran’s chi-square test and the I^2^ statistic, and a random-effects model was applied for the network meta-analysis. Despite these methodological efforts, heterogeneity remained difficult to eliminate. To address this issue, the present study prioritized trials with consistent interventions and outcome measures, applied statistical harmonization procedures, and performed sensitivity analyses to enhance the stability of the results, although residual heterogeneity persists.

Fourth, the model operated under the simplifying assumption of a linear recovery in sleep function to a normal health state over the 60-day timeframe. The structure was justified by clinical guideline recommendations and the maximum treatment duration reported in the included studies. While this provides a standardized and tractable framework for comparison and serves as a practical approximation of the average treatment effect, it may not fully capture the heterogeneity of individual patient recovery patterns. To test the impact of this structural assumption, scenario analyses were conducted, such as extending the recovery duration to 90 days or assuming only 70% of patients achieved full recovery. As presented in the results, the cost-effectiveness rankings were unchanged under these alternative scenarios. The absence of individual-level trajectory data precluded a more in-depth sensitivity analysis of this structural assumption and the exploration of specific alternative recovery patterns (e.g., non-linear curves). Therefore, future studies incorporating more granular individual-level data are warranted to refine these estimates.

On the other hand, this study was conducted within a short-term 60-day analytical framework, which limits the applicability of its conclusions to long-term management. Although the TM + BZDs combination demonstrated the most favorable cost-effectiveness within this time horizon, this finding should not be directly extrapolated to the long-term treatment of insomnia. Long-term use of BZDs carries substantial risks, including dependence, tolerance, and potential cognitive impairment (Liu et al., 2017)), which, along with their potential to increase healthcare costs, were not incorporated into the current model. Therefore, the findings provide useful evidence to inform initial treatment selection. For patients requiring long-term maintenance therapy and to better account for BZDs-related risks, future studies should develop long-term models for further evaluation.

Fifth, the analysis adopted the healthcare system perspective recommended by the China Guidelines for Pharmacoeconomic Evaluations (Liu G. E., 2020), focusing primarily on drug acquisition costs. Consequently, indirect costs (e.g., productivity losses) and the potential transient disutility of mild adverse events were not incorporated, which may result in conservative cost-effectiveness estimates from a broader societal perspective. Future studies that adopt a societal perspective and incorporate individual-level data would be better positioned to comprehensively capture these excluded costs and heterogeneous outcomes. Moreover, given that the short-term model and lack of utility data limited the ability to quantify minor side-effects, longer-term studies should employ dedicated sensitivity analyses to explore their impact of disutility.

Finally, the generalizability of the findings was inherently constrained by their derivation from the Chinese healthcare context. Differences in drug pricing, reimbursement policies, and clinical practices across health systems could significantly alter cost-effectiveness outcomes. Nevertheless, this study provided a valuable economic evaluation framework and reference for other systems considering integration of herbal pharmacotherapies, pending context-specific validation with local data.

### 4.2 Implications for patients, policymakers, and clinical practice

This study provided valuable insights for multiple stakeholders. For patients, the combination of TM + BZDs presented a cost-effective solution, alleviating financial burden while enhancing sleep quality. However, shared decision-making should consider individual preferences, treatment expectations, and out-of-pocket costs.

For policymakers, this analysis offers an economic evidence base to optimize the use of cost-effective CCPPs already covered by insurance, such as TM, and to advance resource allocation. To ensure that economic efficiency translates into equitable health outcomes, policymakers should also address potential implementation challenges. These include regional price disparities and prescriber biases that might limit real-world accessibility, particularly in rural or low-income populations. Future resource allocation frameworks could be strengthened by adopting multi-criteria decision-analysis frameworks that explicitly balance cost-effectiveness with equity considerations, ensuring that evaluations account for both economic efficiency and social justice. In addition, further pharmacoeconomic encompassing of a broader range of CCPPs are needed to complement this evidence base, which can inform the ongoing optimization and expansion of the national insurance formulary.

For clinicians, the findings reinforced TM’s economic advantage in insomnia treatment, encouraging its integration into standardized protocols. Nevertheless, personalized prescribing remained essential. Clinicians should adapt treatment regimens based on disease severity, socioeconomic factors, and patient preferences, while addressing adherence challenges through patient education.

### 4.3 Future research directions

In order to advance the pharmacoeconomics of CCPPs in the treatment of primary insomnia, four essential research directions should be prioritized. First, the integration of diverse real-world data from various regions and cities across China is crucial for validating the long-term efficacy and cost-effectiveness of CCPPs across different populations, including elderly and rural subgroups. This would address the limited generalizability of randomized controlled trials (RCTs), which often fail to capture China’s complex clinical and socioeconomic variables. Second, future pharmacovigilance studies and pragmatic trials are needed to directly monitor and clarify the safety profiles and potential herb-drug interactions of CCPPs within complex real-world populations, where comorbidities and concomitant medications are common. Elucidating the clinical manifestations of these interactions is a critical step toward defining their precise pharmacological mechanisms. Third, societal benefits should be quantified more comprehensively, not only by considering healthcare costs but also by assessing productivity improvements (e.g., reduced absenteeism in the workforce), mental health outcomes (e.g., reductions in depression or anxiety linked to sleep enhancement), and public safety benefits (e.g., fewer accidents due to enhanced alertness). These broader measures of societal impact will offer a more holistic understanding of the benefits of CCPPs, aligning with the multi-dimensional approach of TCM, which considers both physical and mental wellbeing. Fourth, an equity-focused framework should be incorporated, using tools such as social welfare functions (SWF) and concentration indices, to ensure that the treatment benefits do not disproportionately favor high-income or urban populations. Future real-world data studies should be designed to conduct subgroup analyses explicitly stratified by socioeconomic status (e.g., income, education) or geographic region (urban, suburban, rural), and to generate empirical evidence on the magnitude of variation in cost-effectiveness across subpopulations. This evidence is crucial for informing more equitable reimbursement policies, such as tiered copayment structures that ensure access for lower-income groups, thereby supporting more equity-sensitive policy decisions. Lastly, advanced dynamic modeling techniques should be employed to incorporate longitudinal data on relapse risks and nonlinear recovery trajectories. These models would allow for the integration of emerging therapeutic options, such as digital cognitive behavioral therapy (CBT), alongside traditional CCPPs. This would enhance the predictive accuracy of treatment outcomes and provide more nuanced insights into the long-term impact of insomnia interventions.

Furthermore, future studies should focus on the development of TCM-specific health utility scales that capture the unique therapeutic effects and health outcomes associated with CCPPs. These scales would address the complexity of insomnia and reflect the multifaceted nature of TCM treatments, ultimately improving the measurement of health-related quality of life in the context of CCPPs-based therapies.

In summary, these research directions are essential for bridging existing evidence gaps and facilitating more informed decisions regarding resource allocation, ensuring that CCPPs’ clinical efficacy, economic efficiency, and social equity are adequately addressed in the treatment of primary insomnia.

## 5 Conclusion

This study evaluated the cost-effectiveness of four CCPPs (Tian Meng Oral Liquid/Capsules, Shen-Qi-Wu-Wei-Zi Tablets, Wu Ling Capsules, and Bai-Le-Mian Capsules) for primary insomnia from a healthcare perspective. At a WTP threshold of ¥89,358.00 per QALY, the combination of TM and BZDs, within the 60-day time horizon of this analysis, ranked highest in terms of cost-effectiveness, followed by WL, BLM, and SQWWZ. When the WTP threshold exceeded ¥5,897.63 per QALY, the probability that TM + BZDs was more cost-effective than WL increased. Likewise, when the WTP threshold was above ¥19,658.76 per QALY, the probability that BLM was more cost-effective than SQWWZ became greater. Limitations, such as data heterogeneity and model assumptions, highlighted the need for further methodological improvements. Future research should adopt a societal perspective and incorporate individual-level data over longer time horizons to enable more comprehensive cost-utility evaluations, including the disutility of adverse events. Beyond methodological refinement, studies should also focus on collecting real-world datasets from various regions in China, developing TCM-specific health utility scales, and informing equity-focused reimbursement policies to fully address the efficacy, efficiency, and social equity of CCPPs in insomnia treatment.

## Abbreviations

CCPPs, Commercial Chinese polyherbal preparations; PI, primary insomnia; TM, Tian Meng Oral Liquid/Capsules; SQWWZ, Shen-Qi-Wu-Wei-Zi Tablets; WL, Wu Ling Capsules; BLM, Bai-Le-Mian Capsules; BZDs, benzodiazepines; sBZRAs, selective benzodiazepine receptor agonist; NMA, network meta-analysis; PSQI, Pittsburgh sleep quality index; QALY, quality-adjusted life year; CEA, cost-effectiveness analysis; ICER, incremental cost-effectiveness ratio; WTP, willingness-to-pay; DSA, deterministic sensitivity analysis; PSA, probabilistic sensitivity analysis; CEAC, cost-effectiveness acceptability curves; RCT, randomized controlled trials; RR, relative risk; MD, mean difference; CNY, Chinese Yuan.
