# Supplementary material for: Cost-effectiveness analysis of commercial Chinese polyherbal preparations for primary insomnia: based on network meta-analysis
Source: Front Pharmacol. 2025 Dec 8;16:1682173. doi: 10.3389/fphar.2025.1682173 (PMC12722917; doi:10.3389/fphar.2025.1682173)
Supplement: Supplementary file 1 [file Supplementaryfile1.docx]

***Supplementary Materials***

**Contents**

[Table S1. Detailed Preparation Methods and Technical Parameters of the Included CCPPs 2](#_Toc213777846)

[Table S2. CHEERS 2022 Checklist 5](#_Toc213777847)

[Table S3. Basic Characteristics of Included Studies in the Network Meta-analysis 8](#_Toc213777848)

[Table S4. League Table of PSQI Scores 10](#_Toc213777849)

[Table S5. Risk of Bias Assessment Results for Included Studies 11](#_Toc213777850)

[Table S6. Cost-effectiveness results under alternative scenario analyses. 13](#_Toc213777851)

[Figure S1. Schematic Representation of the Two-Phase QALY Estimation Model 16](#_Toc213777852)

[Figure S2. PRISMA flow diagram of literature screening process 17](#_Toc213777853)

### ****Table S1**. **Detailed Preparation Methods and Technical Parameters of the Included CCPPs****

| **Name** | **Formulation^&^** | **Manufacturer** | **Specification** | **Approval Number** | **Preparation Method^#^** |
| --- | --- | --- | --- | --- | --- |
| Wu Ling Capsules (WL) | Wuling Fungus Powder (330g) | Zhejiang Jolly Pharmaceutical Co., Ltd. | 0.33g per capsule | National Drug Approval  Z19990048 | 330g of Wuling fungus powder was filled into capsules to produce 1000 capsules. |
| Tian Meng Oral Liquid/Capsules (TM) | *Acanthopanax senticosus* (Rupr. & Maxim.) Harms (53g), *Polygonatum sibiricum* Redouté (67g), Bombycidae (13g), *Morus alba* L. (33g), *Codonopsis pilosula* Nannf. (40g), *Astragalus mongholicus* Bunge (40g), *Wurfbainia villosa* (Lour.) Škorničk. & A.D.Poulsen (5g), *Lycium barbarum* L. (40g), *Crataegus pinnatifida* Bunge (160g), Prepared Root of *Rehmannia chinensis* Libosch. ex Fisch. & C.A.Mey. (27g), *Epimedium brevicornu* Maxim. (27g), *Citrus reticulata* Blanco (27g), *Poria cocos* (Schw.)Wolf (27g), *Strychnos nux-vomica* L. (1.3g), *Pinellia ternata* (Thunb.) Makino (27g), *Alisma plantago-aquatica subsp. orientale* (Sam.) Sam. (40g), *Dioscorea oppositifolia* L. (27g) | Rongchang Pharmaceutical (Zibo) Co., Ltd. | 10ml per vial / 0.4g per capsule | National Drug Approval  Z20153070/ Z20153057 | 1. **Decoction**: 17 ingredients decocted twice with water (first decoction: 1.5 hours; second decoction: 1 hour). 2. **Filtration & Concentration**: Combined decoctions were filtered and concentrated to a relative density of **1.18–1.20 (at 70°C)**. 3. **Ethanol Precipitation**: Ethanol was added to achieve **65% alcohol content**. The mixture was left to stand. 4. **Supernatant Processing**: The supernatant was collected, ethanol was recovered, and the mixture was concentrated to a relative density of **1.16–1.20 (at 65°C)**. 5. **Refrigeration & Filtration**: Water was added, the mixture was refrigerated, and then filtered. 6. **Finishing**: Sorbate (2g) was added, the volume was adjusted to **1000ml** with water, followed by sterilization and packaging. |
| Bai-Le-Mian Capsules (BLM)* | *Lilium brownii* var. *viridulum* Baker, *Acanthopanax senticosus* (Rupr. & Maxim.) Harms, *Reynoutria multiflora* (Thunb.) Moldenke,  *Albizia julibrissin* var. *julibrissin*, Mother-of-Pearl, Gypsum, *Ziziphus jujuba var. spinosa* (Bunge) Hu ex H.F.Chow, *Poria cocos* (Schw.)Wolf, *Polygala tenella* Willd., *Scrophularia ningpoensis* Hemsl., *Rehmannia glutinosa* (Gaertn.) Libosch. ex DC.,  *Ophiopogon japonicus* (Thunb.) Ker Gawl.,  *Schisandra chinensis* (Turcz.) Baill., *Juncus conglomeratus* L.,  *Salvia miltiorrhiza* Bunge. Excipient: Starch | Yangtze River Pharmaceutical Group [Co., Ltd.](https://synapse.zhihuiya.com/organization/ea0c608e2c0e5a582f64c4af5b1ce584) | 0.27g per capsule | National Drug Approval  Z20020131 | Unavailable |
| Shen-Qi-Wu-Wei-Zi Tablets (SQWWZ) | *Kadsura longipedunculata* Finet & Gagnep.(180g), *Codonopsis pilosula* Nannf. (60g), *Astragalus mongholicus* Bunge (120g), *Ziziphus jujuba var. spinosa* (Bunge) Hu ex H.F.Chow (30g)  Excipient: Starch, Sugar Powder, Talcum powder | Kangxian Duyiwei Biopharmaceutical Co., Ltd. | 0.26g per tablet | National Drug Approval  Z20103006 | 1. **Powder Preparation**: 60g of *Codonopsis pilosula* Nannf. root and a portion of *Kadsura longipedunculata* Finet & Gagnep. were ground into powder. 2. **Macerations**: - *Astragalus mongholicus* Bunge: macerated with **45% ethanol** for 24 hours. - Roasted *Ziziphus jujuba* var. *spinosa* (Bunge) Hu ex H.F.Chow seed: macerated with **70% ethanol** for 24 hours. - Remaining *Kadsura longipedunculata* Finet & Gagnep.: macerated with **60% ethanol** for 24 hours. 3. **Percolate Processing**: Percolates were collected, ethanol was recovered, and the mixture was concentrated. 4. **Tableting**: The concentrated extract was mixed with the prepared powder and excipients, granulated, dried, and compressed into **1000 tablets**. |

*Dosage details for Bai-Le-Mian Capsules’ composition are unavailable; &The botanical components in the formulations have all been verified against The World Flora Online ([https://www.worldfloraonline.org/)[2025-2-25](https://www.worldfloraonline.org/)%5b2025-2-25)]; #Preparation methods sourced from Chinese Pharmacopoeia 2020 Edition(Chinese Pharmacopoeia Commission, 2020) and Yaozhi Database (<https://www.yaozh.com>).

### ****Table S2**. **CHEERS 2022 Checklist****

| **Topic** | **No.** | **Item** | **Location where item is reported** |
| --- | --- | --- | --- |
| **Title** |  |  |  |
|  | 1 | Identify the study as an economic evaluation and specify the interventions being compared. | Title |
| **Abstract** |  |  |  |
|  | 2 | Provide a structured summary that highlights context, key methods, results, and alternative analyses. | Abstract |
| **Introduction** |  |  |  |
| **Background and objectives** | 3 | Give the context for the study, the study question, and its practical relevance for decision making in policy or practice. | Introduction, Fourth Paragraph |
| **Methods** |  |  |  |
| **Health economic analysis plan** | 4 | Indicate whether a health economic analysis plan was developed and where available. | Methods, 2.3.1 Overview |
| **Study population** | 5 | Describe characteristics of the study population (such as age range, demographics, socioeconomic, or clinical characteristics). | Methods, 2.2.2 Inclusion and Exclusion Criteria |
| **Setting and location** | 6 | Provide relevant contextual information that may influence findings. | Methods, 2.2.2 Inclusion and Exclusion Criteria |
| **Comparators** | 7 | Describe the interventions or strategies being compared and why chosen. | Methods, 2.2.2 Inclusion and Exclusion Criteria |
| **Perspective** | 8 | State the perspective(s) adopted by the study and why chosen. | Methods, 2.3.1 Overview |
| **Time horizon** | 9 | State the time horizon for the study and why appropriate. | Methods, 2.3.1 Overview |
| **Discount rate** | 10 | Report the discount rate(s) and reason chosen. | Methods, 2.3.1 Overview |
| **Selection of outcomes** | 11 | Describe what outcomes were used as the measure(s) of benefit(s) and harm(s). | Methods, 2.2.2 Inclusion and Exclusion Criteria |
| **Measurement of outcomes** | 12 | Describe how outcomes used to capture benefit(s) and harm(s) were measured. | Methods, 2.2.2 Inclusion and Exclusion Criteria |
| **Valuation of outcomes** | 13 | Describe the population and methods used to measure and value outcomes. | Methods, 2.2.2 Inclusion and Exclusion Criteria |
| **Measurement and valuation of resources and costs** | 14 | Describe how costs were valued. | Methods, 2.3.3 Costs |
| **Currency, price date, and conversion** | 15 | Report the dates of the estimated resource quantities and unit costs, plus the currency and year of conversion. | Methods, 2.3.3 Costs |
| **Rationale and description of model** | 16 | If modelling is used, describe in detail and why used. Report if the model is publicly available and where it can be accessed. | Methods, 2.3.2 Model |
| **Analytics and assumptions** | 17 | Describe any methods for analysing or statistically transforming data, any extrapolation methods, and approaches for validating any model used. | Methods, 2.3.2 Model |
| **Characterising heterogeneity** | 18 | Describe any methods used for estimating how the results of the study vary for subgroups. | Methods , 2.3.5 Sensitivity Analysis |
| **Characterising distributional effects** | 19 | Describe how impacts are distributed across different individuals or adjustments made to reflect priority populations. | Methods , 2.3.5 Sensitivity Analysis |
| **Characterising uncertainty** | 20 | Describe methods to characterise any sources of uncertainty in the analysis. | Methods , 2.3.5 Sensitivity Analysis |
| **Approach to engagement with patients and others affected by the study** | 21 | Describe any approaches to engage patients or service recipients, the general public, communities, or stakeholders (such as clinicians or payers) in the design of the study. | Not applicable |
| **Results** |  |  |  |
| **Study parameters** | 22 | Report all analytic inputs (such as values, ranges, references) including uncertainty or distributional assumptions. | Results, 3.2.1 Base-Case Analysis |
| **Summary of main results** | 23 | Report the mean values for the main categories of costs and outcomes of interest and summarise them in the most appropriate overall measure. | Results, 3.2.1 Base-Case Analysis |
| **Effect of uncertainty** | 24 | Describe how uncertainty about analytic judgments, inputs, or projections affect findings. Report the effect of choice of discount rate and time horizon, if applicable. | Results, 3.2.2 Sensitivity Analysis |
| **Effect of engagement with patients and others affected by the study** | 25 | Report on any difference patient/service recipient, general public, community, or stakeholder involvement made to the approach or findings of the study | Not applicable |
| **Discussion** |  |  |  |
| **Study findings, limitations, generalisability, and current knowledge** | 26 | Report key findings, limitations, ethical or equity considerations not captured, and how these could affect patients, policy, or practice. | Discussion, 4.1 Study Limitations and 4.2 Implications for Patients, Policymakers, and Clinical Practice |
| **Other relevant information** |  |  |  |
| **Source of funding** | 27 | Describe how the study was funded and any role of the funder in the identification, design, conduct, and reporting of the analysis | Not Anonymized |
| **Conflicts of interest** | 28 | Report authors conflicts of interest according to journal or International Committee of Medical Journal Editors requirements. | Not Anonymized |

 From: Husereau D, Drummond M, Augustovski F, et al. Consolidated Health Economic Evaluation Reporting Standards 2022 (CHEERS 2022) Explanation and Elaboration: A Report of the ISPOR CHEERS II Good Practices Task Force. Value Health 2022;25.

### ****Table S3**. **Basic Characteristics of Included Studies in the Network Meta-analysis****

| **Study ID** | **Intervention** | **Sample Size (n)** | **Duration (d)** | **PSQI Score** | | | | | | **Dosage** | **Administration Regimen** | **Study Weight** | **Daily Dose / Drug 1** | **Daily Dose / Drug 2** | **Weighted Daily Dose / Drug 1** | **Weighted Daily Dose / Drug 2** | **Weighted Duration (d)** |
| --- | --- | --- | --- | --- | --- | --- | --- | --- | --- | --- | --- | --- | --- | --- | --- | --- | --- |
|  |  |  |  | **Baseline Mean** | **SD** | **Endpoint Mean** | **SD** | **Reduction Value** | **SD** |  |  |  |  |  |  |  |  |
| Feng XD 2008 | WL | 25 | 60 | 12.30 | 1.29 | 7.20 | 0.78 | 5.10 | 1.13 | 0.33 g/cap | 3 caps tid | 7% | 9 |  | 8.808 |  | 30.13333 |
| Huang XY 2011 | WL | 50 | 28 | 14.25 | 0.41 | 9.56 | 0.36 | 4.69 | 0.39 | 0.33 g/cap | 3 caps tid | 13% | 9 |  |  |  |  |
| Jin X 2012 | WL | 24 | 28 | 12.29 | 1.24 | 4.48 | 2.96 | 7.81 | 2.57 | 0.33 g/cap | 3 caps tid | 6% | 6 |  |  |  |  |
| Song XH 2010 | WL | 94 | 28 | 11.78 | 2.80 | 7.53 | 3.11 | 4.25 | 2.97 | 0.33 g/cap | 3 caps tid | 25% | 9 |  |  |  |  |
| Wang J 2021 | WL | 49 | 28 | 19.94 | 2.30 | 7.58 | 0.90 | 12.36 | 2.00 | 0.33 g/cap | 3 caps tid | 13% | 9 |  |  |  |  |
| Yan Lin 2013 | WL | 94 | 28 | 11.78 | 2.80 | 7.53 | 3.11 | 4.25 | 2.97 | 0.33 g/cap | 3 caps tid | 25% | 9 |  |  |  |  |
| Zhang SJ 2017 | WL | 39 | 28 | 10.02 | 3.63 | 7.45 | 2.42 | 2.57 | 3.20 | 0.33 g/cap | 3 caps tid | 10% | 9 |  |  |  |  |
| Chen H 2002 | BLM | 30 | 56 | 13.70 | 2.40 | 5.70 | 1.00 | 8.00 | 1.83 | 0.27g/cap | 4 caps bid | 16% | 8 |  | 8 |  | 35.97927 |
| Jin X 2012 | BLM | 24 | 28 | 12.07 | 2.13 | 11.83 | 1.96 | 0.24 | 2.05 | 0.27g/cap | 4 caps bid | 12% | 8 |  |  |  |  |
| Lin YW 2017 | BLM | 42 | 65 | 14.62 | 2.29 | 7.34 | 1.83 | 7.28 | 2.10 | 0.27g/cap | 4 caps bid | 34% | 8 |  |  |  |  |
| Mai J 2009 | BLM | 30 | 21 | 19.40 | 4.12 | 6.20 | 2.11 | 13.20 | 3.57 | 0.27g/cap | 4 caps bid | 16% | 8 |  |  |  |  |
| Zhang SJ 2017 | BLM | 44 | 28 | 10.10 | 3.34 | 6.14 | 2.11 | 3.96 | 2.93 | 0.27g/cap | 4 caps bid | 23% | 8 |  |  |  |  |
| Zeng ZL2014 | SQWWZ | 50 | 56 | 12.80 | 2.50 | 9.30 | 2.20 | 3.50 | 2.36 | 0.25g/tab | 5 tabs tid | 49% | 15 |  | 15 |  | 56 |
| Yu ZA 2009 | SQWWZ | 53 | 56 | 13.50 | 2.40 | 9.80 | 2.30 | 3.70 | 2.35 | 0.25g/tab | 5 tabs tid | 51% | 15 |  |  |  |  |
| Wang XY 2016 | TM + BZD (Oxazepam) | 40 | 28 | 14.52 | 3.63 | 6.12 | 2.47 | 8.40 | 3.21 | 10ml/vial +15mg/tab | TM: 10 ml bid, Oxazepam: 15–30 mg qd | 29% | 2 | 1.5 | 2 | 1.5 | 28 |
| Tong JY 2019 | TM + BZD (Oxazepam) | 62 | 28 | 14.26 | 3.52 | 8.53 | 2.84 | 5.73 | 3.23 | 10ml/vial +15mg/tab | TM: 10 ml bid, Oxazepam: 15–30 mg qd | 45% | 2 | 1.5 |  |  |  |
| Li SY2012 | TM + BZD (Oxazepam) | 35 | 28 | 13.66 | 2.92 | 6.51 | 1.40 | 7.15 | 2.53 | 0.4g/cap +15mg/tab | TM: 3 caps bid, Oxazepam: 15–30 mg qd | 26% | 6 | 1.5 | 6 | 1.5 | 28 |

**PSQI: Pittsburgh sleep quality index; SD: Standard Deviation; WL: Wu Ling Capsules; BLM: Bai-Le-Mian Capsules; SQWWZ: Shen-Qi-Wu-Wei-Zi Tablets; TM: Tian Meng Oral Liquid/Capsules; BZDs: benzodiazepines; cap: capsule; tab: tablet, vial: small bottle for liquid medication; mg: milligram; ml: milliliter; qd: once daily (from Latin: quaque die); bid: twice daily (from Latin: bis in die); tid: three times daily (from Latin: ter in die);**

### ****Table S4. League Table of PSQI Scores****

| BLM |  |  |  |  |  |
| --- | --- | --- | --- | --- | --- |
| 1.05 ( -1.80 to 3.90) | WL |  |  |  |  |
| 1.84 ( -2.95 to 6.63) | 0.79 ( -3.64 to 5.22) | SQWWZ |  |  |  |
| 1.67 ( -2.64 to 5.99) | 0.63 ( -3.29 to 4.55) | -0.16 ( -4.97 to 4.65) | TM+BZDs |  |  |
| -1.61 ( -4.65 to 1.42) | -2.66 ( -5.10 to -0.22) | -3.45 ( -7.15 to 0.25) | -3.29 ( -6.36 to -0.22) | BZDs |  |
| -1.70 ( -5.26 to 1.87) | -2.74 ( -5.91 to 0.42) | -3.53 ( -8.83 to 1.76) | -3.37 ( -8.24 to 1.50) | -0.08 ( -3.87 to 3.70) | Placebo |

BLM: Bai-Le-Mian Capsules; WL: Wu Ling Capsules; SQWWZ: Shen-Qi-Wu-Wei-Zi Tablets; TM: Tian Meng Oral Liquid/Capsules; BZDs: benzodiazepines

### ****Table S5. Risk of Bias Assessment Results for Included Studies****

| **No.** | **Study ID** | **Experimental Intervention** | **Comparator Intervention** | **Random sequence generation** | **Allocation concealment** | **Blinding of participants and personnel** | **Blinding of outcome assessment** | **Incomplete outcome data** | **Selective outcome reporting** |
| --- | --- | --- | --- | --- | --- | --- | --- | --- | --- |
| 1 | Feng XD 2008 | WL | Alprazolam Tablets | PY | PN | PN | PN | DY | DY |
| 2 | Huang XY 2011 | WL | Diazepam | PY | PN | PN | PN | DY | DY |
| 3 | Jin X 2012 | WL | BLM | PY | PN | PN | PN | DY | DY |
| 4 | Song XH 2010 | WL | Placebo | DY | DY | DY | DY | PY | DY |
| 5 | Wang J 2021 | WL | Estazolam Tablets | DY | PN | DY | PN | DY | DY |
| 6 | Yan L 2013 | WL | Placebo | DY | DY | DY | DY | PY | DY |
| 7 | Chen H 2002 | BLM | Zolpidem Tablets | PY | PN | PN | PN | DY | DY |
| 8 | Lin YW 2017 | BLM | Placebo | DY | PN | PN | PN | DY | DY |
| 9 | Mai J 2009 | BLM | Triazolam Tablets | PY | PN | PN | PN | DY | DY |
| 10 | Zhang SJ 2017 | BLM | WL / Oxazepam | DY | PN | PN | PN | DY | DY |
| 11 | Zeng ZL 2014 | SQWWZ | Alprazolam Tablets | PY | PN | PN | PN | DY | DY |
| 12 | Yu ZA 2009 | SQWWZ | Alprazolam Tablets | PN | PN | PN | PN | DY | DY |
| 13 | Wang XY 2016 | TM+BZDs | Oxazepam | PY | PN | DY | PN | DY | DY |
| 14 | Tong JY 2019 | TM+BZDs | Oxazepam | DY | PN | PN | PN | DY | DY |
| 15 | Li SY 2012 | TM+BZDs | Oxazepam | PN | PN | PN | PN | DY | DY |

PY: Probably yes; PN: Probably no; DY: Definitely yes.

### ****Table S6. Cost-effectiveness results under alternative scenario analyses.****

| **Scenario** | **Regimen** | **QALY** | **Costs(¥)** | **ΔQALY** | **ΔCost (¥)** | **ICER** | **WTP (¥)** | **Dominance** |
| --- | --- | --- | --- | --- | --- | --- | --- | --- |
|  |  |  |  |  |  | **(¥/QALY)** |  |  |
| Base Case | TM + BZD | 0.119 | 287.812 | 0.001 | 5.359 | 4350.317 | 89358 | Dominant |
|  | vs. WL | 0.118 | 282.453 |  |  |  |  |  |
|  | TM + BZD | 0.119 | 287.812 | 0.013 | -41.810 | -3220.957 | 89358 | Dominant |
|  | vs. SQWWZ | 0.106 | 329.622 |  |  |  |  |  |
|  | TM + BZD | 0.119 | 287.812 | 0.004 | -213.361 | -49031.042 | 89358 | Dominant |
|  | vs. BLM | 0.114 | 501.173 |  |  |  |  |  |
|  | WL | 0.118 | 282.453 | 0.012 | -47.169 | -4014.764 | 89358 | Dominant |
|  | vs. SQWWZ | 0.106 | 329.622 |  |  |  |  |  |
|  | WL | 0.118 | 282.453 | 0.003 | -218.720 | -70108.062 | 89358 | Dominant |
|  | vs. BLM | 0.114 | 501.173 |  |  |  |  |  |
|  | SQWWZ | 0.106 | 329.622 | -0.009 | -171.551 | 19880.658 | 89358 | Dominated |
|  | vs. BLM | 0.114 | 501.173 |  |  |  |  |  |
| Prolonged Recovery (90d) | TM + BZD | 0.185 | 287.812 | 0.001 | 5.359 | 3966.790 | 89358 | Dominant |
|  | vs. WL | 0.183 | 282.453 |  |  |  |  |  |
|  | TM + BZD | 0.185 | 287.812 | 0.013 | -41.810 | -3228.480 | 89358 | Dominant |
|  | vs. SQWWZ | 0.172 | 329.622 |  |  |  |  |  |
|  | TM + BZD | 0.185 | 287.812 | 0.005 | -213.361 | -45696.013 | 89358 | Dominant |
|  | vs. BLM | 0.180 | 501.173 |  |  |  |  |  |
|  | WL | 0.183 | 282.453 | 0.012 | -47.169 | -4066.454 | 89358 | Dominant |
|  | vs. SQWWZ | 0.172 | 329.622 |  |  |  |  |  |
|  | WL | 0.183 | 282.453 | 0.003 | -218.720 | -65914.290 | 89358 | Dominant |
|  | vs. BLM | 0.180 | 501.173 |  |  |  |  |  |
|  | SQWWZ | 0.172 | 329.622 | -0.008 | -171.551 | 20715.704 | 89358 | Dominated |
|  | vs. BLM | 0.180 | 501.173 |  |  |  |  |  |
| Incomplete Recovery (70%) | TM + BZD | 0.114 | 287.812 | 0.001 | 0.001 | 5.359 | 89358 | Dominant |
|  | vs. WL | 0.113 | 282.453 |  |  |  |  |  |
|  | TM + BZD | 0.114 | 287.812 | 0.009 | 0.009 | -41.810 | 89358 | Dominant |
|  | vs. SQWWZ | 0.105 | 329.622 |  |  |  |  |  |
|  | TM + BZD | 0.114 | 287.812 | 0.003 | 0.003 | -213.361 | 89358 | Dominant |
|  | vs. BLM | 0.111 | 501.173 |  |  |  |  |  |
|  | WL | 0.113 | 282.453 | 0.008 | 0.008 | -47.169 | 89358 | Dominant |
|  | vs. SQWWZ | 0.105 | 329.622 |  |  |  |  |  |
|  | WL | 0.113 | 282.453 | 0.002 | 0.002 | -218.720 | 89358 | Dominant |
|  | vs. BLM | 0.111 | 501.173 |  |  |  |  |  |
|  | SQWWZ | 0.105 | 329.622 | -0.006 | -0.006 | -171.551 | 89358 | Dominated |
|  | vs. BLM | 0.111 | 501.173 |  |  |  |  |  |
| With Consultation Costs | TM + BZD | 0.119 | 367.812 | 0.001 | -14.641 | -11886.097 | 89358 | Dominant |
|  | vs. WL | 0.118 | 382.453 |  |  |  |  |  |
|  | TM + BZD | 0.119 | 367.812 | 0.013 | -101.810 | -7843.238 | 89358 | Dominant |
|  | vs. SQWWZ | 0.106 | 469.622 |  |  |  |  |  |
|  | TM + BZD | 0.119 | 367.812 | 0.004 | -233.361 | -53627.102 | 89358 | Dominant |
|  | vs. BLM | 0.114 | 601.173 |  |  |  |  |  |
|  | WL | 0.118 | 382.453 | 0.012 | -87.169 | -7419.366 | 89358 | Dominant |
|  | vs. SQWWZ | 0.106 | 469.622 |  |  |  |  |  |
|  | WL | 0.118 | 382.453 | 0.003 | -218.720 | -70108.062 | 89358 | Dominant |
|  | vs. BLM | 0.114 | 601.173 |  |  |  |  |  |
|  | SQWWZ | 0.106 | 469.622 | -0.009 | -131.551 | 15245.153 | 89358 | Dominated |
|  | vs. BLM | 0.114 | 601.173 |  |  |  |  |  |

QALY: quality-adjusted life year; ICER: incremental cost-effectiveness ratio; WTP: willingness-to-pay; TM: Tian Meng Oral Liquid/Capsules; BZDs: benzodiazepines; WL: Wu Ling Capsules; SQWWZ: Shen-Qi-Wu-Wei-Zi Tablets; BLM: Bai-Le-Mian Capsules

**
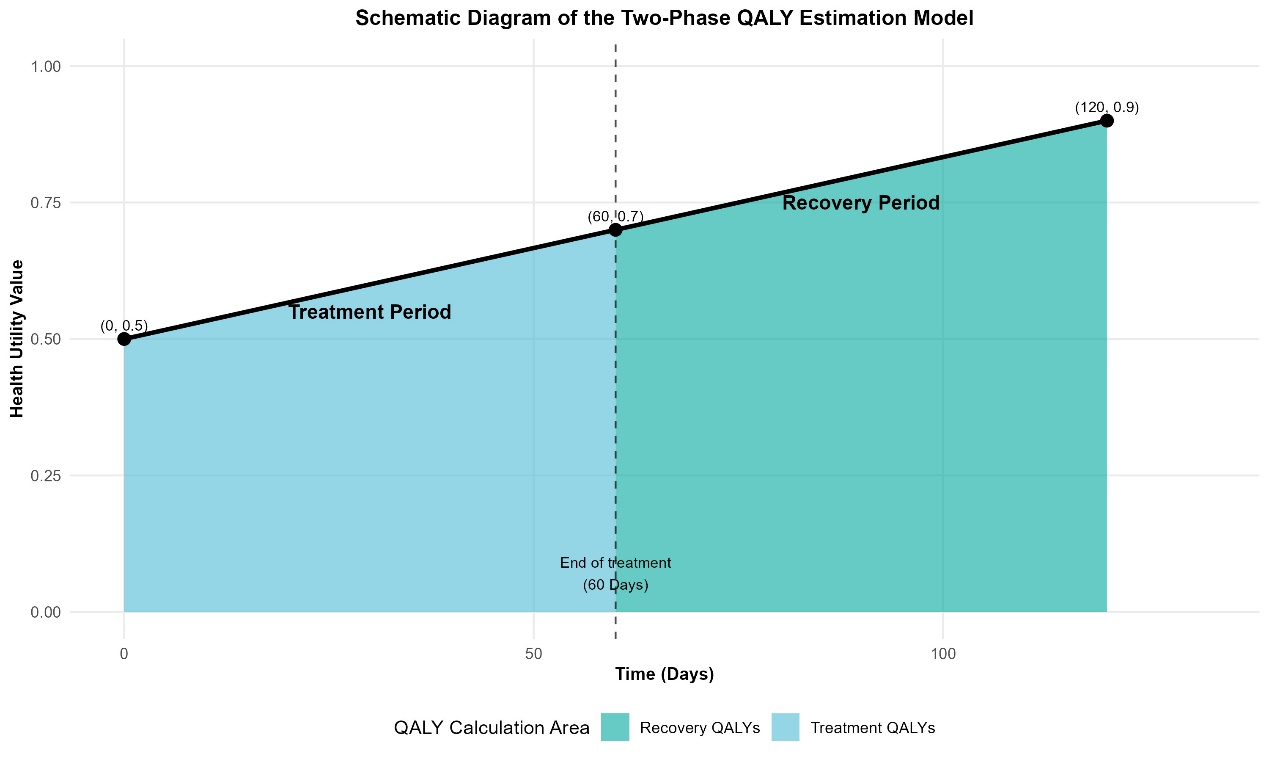
**

### ****Figure S1. Schematic Representation of the Two-Phase QALY Estimation Model****

**This diagram illustrates the conceptual framework for estimating Quality-Adjusted Life Years (QALYs) in the cost-effectiveness analysis, corresponding to Formula 4 in the main text. The model divides the follow-up period into two phases:**

**Treatment Period (blue area): From baseline (day 0) to the end of active treatment (Treatment Duration). During this phase, health utility improves linearly from the baseline utility (Utility_baseline) to the post-treatment utility (Utility_post-treatment). QALYs in this period are calculated as the area under the curve using the trapezoidal rule: (Utility_baseline + Utility_post-treatment) × Treatment Duration ÷ 2 ÷ 365.**

**Recovery Period (green area): From the end of active treatment to the end of the study observation period (Study Duration). During this phase, health utility continues to improve linearly from the post-treatment utility (Utility_post-treatment) to the normal health state utility (Utility_normal). QALYs in this period are calculated as: (Utility_post-treatment + Utility_normal) × (Study Duration - Treatment Duration) ÷ 2 ÷ 365.**

**The total QALYs for each intervention are the sum of the QALYs from both periods. This two-phase approach captures the distinct utility trajectories during active treatment and subsequent recovery, reflecting the clinical course of primary insomnia treatment.**


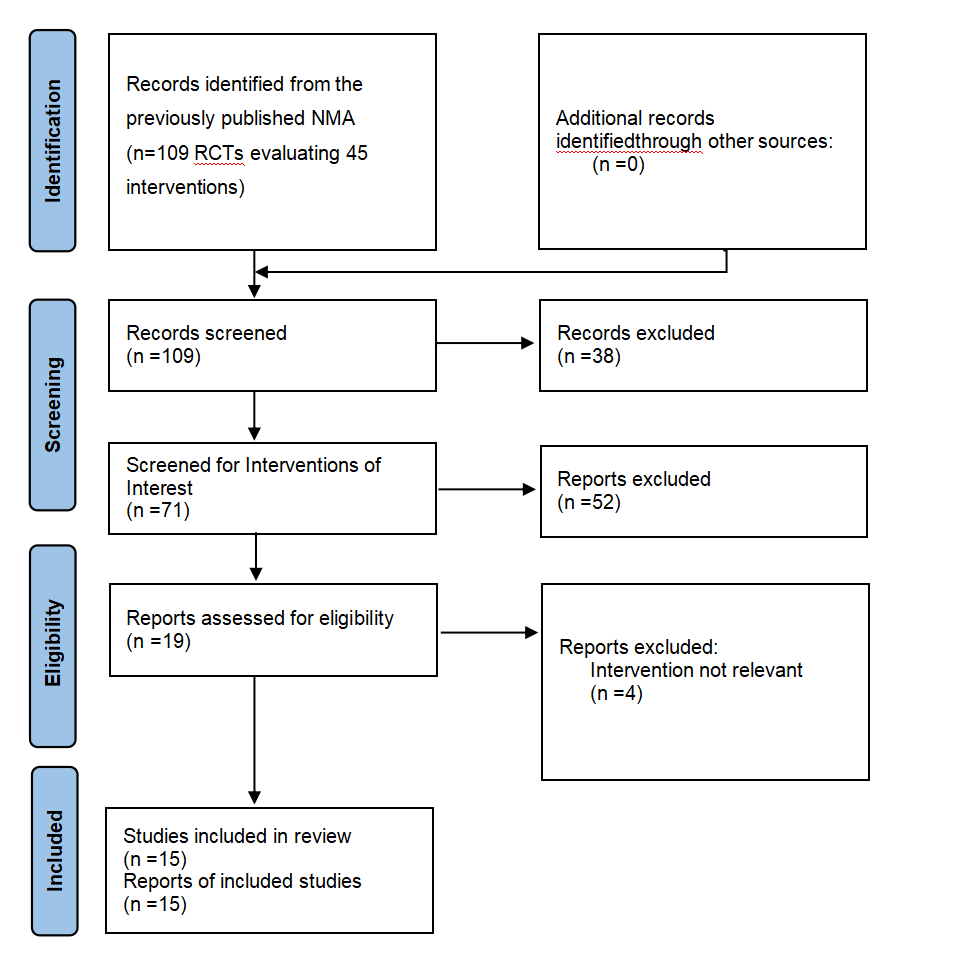


### ****Figure S2. PRISMA flow diagram of literature screening process****
